# Immunomodulatory Roles of Cell Cycle Regulators

**DOI:** 10.3389/fcell.2019.00023

**Published:** 2019-02-26

**Authors:** Phatthamon Laphanuwat, Siwanon Jirawatnotai

**Affiliations:** Department of Pharmacology, Faculty of Medicine Siriraj Hospital, Siriraj Center of Research for Excellence for Systems Pharmacology, Mahidol University, Bangkok, Thailand

**Keywords:** CDKs, cyclins, non-cell cycle functions, innate immune system, adaptive immune system, immunosurveillance

## Abstract

Core cell cycle regulators, including cyclin-dependent kinases (CDKs), cyclins, and cyclin-dependent kinase inhibitors (CKIs), are known for their well-characterized roles in cell division. Several recent studies have shed light on the roles of these proteins in immune modulation. The development and activation of cells in the immune system take place not only during embryonic development but throughout the life of a multicellular organism. Cell cycle regulators are involved in the development of immune cells, partly as the machinery controlling the expansion and differentiation of the populations of immune cells. In addition, these proteins serve non-cell cycle functions. In this review, we summarize the emerging roles of cell cycle regulators in modulating functions of the immune system and discuss how they may be exploited as therapeutic targets.

## Introduction

Multicellular organisms develop and maintain tissue homeostasis through cell division. Cell division is governed by a group of core proteins called cyclin-dependent kinases (CDKs), cyclins, and cyclin-dependent kinase inhibitors (CKIs), which are negative regulators of CDKs. Growth factors induce the expression of D cyclins (cyclins D1, D2, and D3), which are regarded as molecular links between the cell environment and the core cell cycle machinery. Once induced, D cyclins bind to CDK4 or CDK6 and phosphorylate the pocket proteins pRB, p107, and p130, which bind and regulate E2F transcription factors during the G1 phase of the cell cycle. CDK4/6-dependent phosphorylation of the pocket proteins converts the pocket protein-E2F complex from transcriptional repressor to activator. During late G1, E cyclins (cyclins E1 and E2) become upregulated and activate CDK2, resulting in phosphorylation of various cell cycle-related proteins. During S phase, the induction of cyclin A2, which partners with CDK2 and CDK1, and activation of cyclin B1–CDK1 at the onset of mitosis drive the progression of cells through the remainder of the cell cycle through phosphorylation of a large number of proteins involved in DNA replication, as well as in centrosome and chromosome function. CDK activities are negatively regulated by CKI binding. CKIs have been classified into two families. The Cip/Kip family comprises p21^CIP1^, p27^KIP1^, and p57^KIP2^, and these proteins are inhibitors of CDK2/1. The other family, designated INK4, consists of p16^INK4A^, p15^INK4B^, p18^INK4C^, and p19^INK4D^, which are inhibitors of CDK4/6. More detailed information on these proteins can be found in recent articles (Malumbres, 2014; Hydbring et al., 2016).

In addition to cell cycle regulation in cell division, emerging roles of these proteins in other biological processes have been reported (Lim and Kaldis, 2013; Hydbring et al., 2016). Interestingly, some non-canonical functions of the cell cycle regulators are operating beyond cell-autonomous level. These proteins have recently been shown to be integral regulators of the immune system, highlighting them as essential components of both tissue-tissue and tissue-environmental communications in multicellular organisms.

Immune cells in the bone marrow and thymus undergo repeated cycling as part of their development. In addition, following antigen exposure, mature lymphocytes proliferate rapidly to establish a timely immune response and generate immunological memory. Proliferation also precedes induction of tolerance to soluble antigens. Therefore, it appears that cell proliferation is essential for immune-system function. Cell cycle protein involvement in immune function was initially indicated in experiments in which the genes encoding the cell cycle regulators were disrupted in mice. These knockout experiments demonstrated an essential role of individual cell cycle regulators during immune cell development (Sherr and Roberts, 2004). For example, cyclin D3-deficient mice displayed reduced levels of neutrophil granulocytes in their peripheral blood. The cyclin D3-deficient granulocytes were refractory to granulocyte colony-stimulating factor (G-CSF)-driven expansion (Sicinska et al., 2006). Cyclin D2 was shown to be required for the proliferation of CD5+ B cells and the antigen-dependent B cell clonal expansion (Solvason et al., 2000). These results indicate that the cell cycle regulators may play a major role in the homeostasis and integrity of the immune system.

However, emerging evidence has demonstrated that cell cycle regulators also directly control activities of several types of immune cells. In this review, we discuss the direct immunomodulating roles of cell cycle regulators, in both the innate and adaptive immune systems. Our objectives are to understand this aspect of these proteins systematically, and to reassess these proteins as potential targets for pharmacological treatment of diseases such as cancer or autoimmune diseases.

## Emerging Roles of Cell Cycle Regulators in the Innate Immune Response

Innate immunity serves as the first line of host defense and plays an essential role in preventing infection. Defects in innate immunity are associated with invasive, life-threatening infection. Inappropriate activation of the innate immune system can lead to autoinflammatory states. The cellular components of the innate immune system are neutrophils, monocytes/macrophages, dendritic cells, and innate lymphoid cells. The innate immune system also directs the subsequent development of adaptive immune responses. Its proper function is thus critical for health.

The overall function of cell cycle regulators in the innate immune response appears to be as innate immune facilitators (Matsushime et al., 1991). Cell cycle proteins such as CDKs and CKIs play direct roles in ensuring expansion of the cells in the innate immune system and maintaining balanced innate immune activities.

**FIGURE 1:**
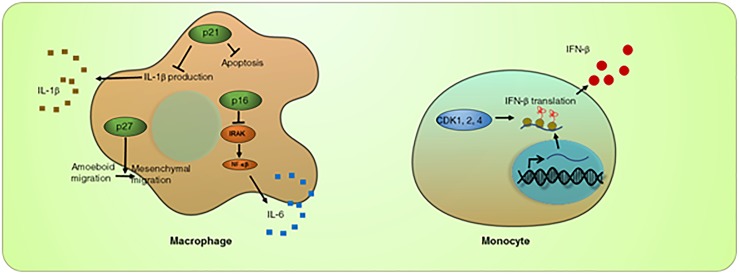
Roles of cell cycle regulators in innate immune response. CDKs play a positive role in IFN-β translation. CDK inhibition leads to reduced IFN-β mRNA in the polysome complex. On the other hand, CDK inhibitor p21^CIP1^ is required to suppress overproduction of IL-1β, and p16^INK4A^ is required to suppress IL-6, in macrophages. p27^KIP1^ has a unique role in promoting macrophage mesenchymal migration, thus facilitating macrophage tumor infiltration.

A direct, non-cell cycle function of CDKs in the innate immune response has been described recently. Following viral infection, innate immune cells such as monocytes produce type I interferon (IFN) to protect the host against viral invasion. Inhibition of CDKs in a monocyte cell line, THP-1, inhibited secretion of type I IFN (IFN-β). Activity of CDK1, 2, and 4 was shown to be essential for the translation of type I IFN mRNA (Cingoz and Goff, 2018). In the absence of CDK activity, caused by a pan-CDK inhibitor (including R547; CDK1, 2, 4 inhibitor, Dinaciclib; CDK2, 5, 1, 9 inhibitor, AZD5438; CDK1, 2, 9 inhibitor, SNS-032; CDK2, 7, 9 inhibitor), IFN-β mRNA is removed from the translating polysome complex, while global translation is not affected. This suggests that CDK activity is specifically required for IFN-β production (Cingoz and Goff, 2018), which in turn initiates immune system activation (Figure 1). These findings require several follow up studies. Since most of the findings in this study relied on pan-CDK inhibitors, genetic experiments in which one or more CDKs are specifically knocked out should be performed to validate the results. It is possible that pan-CDK inhibitors cause immediate cell cycle arrest, indirectly affecting the translation of the IFN mRNA. It is also unclear how CDK kinase activity keeps the IFN-β mRNA in the translating polysome complex. In addition, it is unclear how active CDK/cyclin complexes, known to be in the nucleus, can control cytosolic translation.

Macrophages are multifunctional innate immune cells present in all tissues within the body, awaiting invading pathogens. Macrophages contribute to innate immunity *via* phagocytosis, and its function in innate immunological memory. They engulf and digest cellular debris, foreign substances, microbes, and cancer cells. Macrophages that reside in healthy adult tissues are either derived from circulating monocytes or are established before birth and then maintained during adult life, independent of monocytes (Varol et al., 2015). Macrophages are foremost among cells that present antigens, and thus are essential for initiating the adaptive immune response. In addition, macrophages can play a role as secretory cells, which are vital to the regulation of immune responses and the development of inflammation. They produce a wide array of powerful chemical substances including enzymes, complement proteins, and regulatory factors such as interleukin-1. Colony stimulating factor (CSF), granulocyte-macrophage colony stimulating factor (GM-CSF), VEGF, and interleukin 3 (IL3) act as macrophage expansion factors (Wynn et al., 2013).

Cyclin-dependent kinase inhibitors, such as p21^CIP1^, p27^KIP1^, and p16^INK4A^, have been shown to directly regulate macrophage differentiation and activity (Aderem and Underhill, 1999; Yoshida et al., 2015; Kapellos et al., 2016). Growth factors such as CSF, GM-CSF, and IL-3 induce the PI3K/AKT-dependent upregulation of p21^CIP1^ (Comalada et al., 2004). Through an unknown cell cycle-independent mechanism, the upregulation of p21^CIP1^ protects macrophages from undergoing apoptosis (Comalada et al., 2004). p21^CIP1^ was also shown to restrain macrophage activity to an optimum level; without p21^CIP1^, macrophages overreact when stimulated. Mice deficient in p21^CIP1^ appear to be more susceptible to lipopolysaccharide-induced septic shock, which is associated with increased serum levels of the inflammatory factor IL-1β. Furthermore, p21^CIP1^ deficiency leads to autoinflammatory diseases, such as lupus erythematosus and arthritis (Kong et al., 2007). IL-1β released from macrophages can trigger self-stimulation and activate other immune cells, including neutrophils and monocytes. p21^CIP1^ suppresses IL-1β at both the transcription and pro-protein levels, suggesting a role for p21^CIP1^ in limiting excessive macrophage activation (Scatizzi et al., 2009; Trakala et al., 2009; Figure 1). Macrophage activation is mediated by the transcription factor NF-κB. p21^CIP1^-deficient macrophages correlate with increased NF-κB activity (Trakala et al., 2009). These findings point to p21^CIP1^ as a key regulator of macrophage activity.

p16^INK4A^ also inhibits macrophage activity. Expression of p16^INK4A^ promotes a ubiquitin-dependent degradation of interleukin-1 receptor (IL-1R) associated kinase, which is an inducer for the IL-6 pathway. Thus, forced expression of p16^INK4A^ impaired IL-6 production and inhibited inflammatory cytokine production, leading to a reduction of tissue inflammation (Murakami et al., 2012; Figure 1).

Thus, the CKIs p21^CIP1^ and p16^INK4A^ contribute to maintenance of a balanced response to inflammatory stimuli. Mechanistically, it remains unclear whether the macrophage modulating roles of p21^CIP1^ and p16^INK4A^ are mediated by their CDK-inhibitory activities. Peptide mapping showed that the CDK-binding domain of p21^CIP1^ is sufficient to reduce the secretion of IL-1β (Scatizzi et al., 2009), implying that the CDK activity may be involved; If so, it would be interesting to identify the targeted CDK or CDKs. Interestingly, CDK2, 5, and 7 were identified in a high throughput short interfering RNA screen as positive regulators for TNF-induced NF-κB activity (Choudhary et al., 2011). Thus, it is possible that at least part of the function of p21^CIP1^ is to oppose CDK2 activity in macrophages. In addition, it may be interesting to determine whether inhibition of CDK activity by small molecule CDK inhibitors will phenocopy the overexpression of the CKIs, and whether small molecule CDK inhibitors may be used to manage septic shock and autoinflammatory diseases.

Lastly, p27^KIP1^ was shown to support the anti-tumor activity of macrophages. Macrophage infiltration into tissue is critical in initiating the immune response as well as the inflammatory response. Macrophages use two types of migration: amoeboid and mesenchymal migration. Amoeboid migration is used when migrating through loose tissues, whereas mesenchymal migration is used when migrating into a dense matrix such as a tumor mass. Cytoplasmic p27^KIP1^ suppresses ROCK-mediated amoeboid migration and promotes mesenchymal migration (Gui et al., 2014; Figure 1).

## Roles of Cell Cycle Regulators in the Adaptive Immune Response

The adaptive immune system, or the acquired immune system, creates immunological memory after an initial response to a specific pathogen, and leads to an enhanced response to subsequent encounters with that pathogen. Lymphocytes are the cells that carry out the acquired immune response. Two types of lymphocytes, B cells and T cells, are responsible for carrying out the main classes of adaptive immunity, antibody responses and cell mediated immune response. Similar to innate immune cells, genetic experiments showed that specific cell cycle regulators are essential for the adaptive immune cell numbers. Cyclin D3-CDK6 activity is required to maintain thymocyte numbers; mice deficient in CDK6 or cyclin D3 showed a significant decrease in thymocyte numbers (Sicinska et al., 2003; Malumbres et al., 2004; Hu et al., 2009). In addition, during T cell activation, cell cycle proteins indirectly control lymphocyte activity. T cells that complete more cell cycle rounds during the primary response are more likely to produce effector cytokines upon secondary challenge (Bird et al., 1998; Gett and Hodgkin, 1998). D cyclins link B cell development and activity. Clonal expansion of B cells involves rapid proliferation to form a germinal center (GC). Within the GC, B cells undergo class switching, clonal expansion, and clonal selection to generate a high-affinity humoral immune Ig.

Under several circumstances, cell cycle regulators are believed to link cell division to differentiation. Distinguishing the contributions of these two processes in immune cell activity is not straightforward. A knockout study showed that cyclin D3-deficient mice have a mild reduction of follicular B cells and increased marginal zone B cells. Functionally, cyclin D3-deficient GCs have severe impairment of GC reactions and defective antibody responses (Peled et al., 2010). In the same study, cyclin D3 was shown to be a downstream effector that mediates the GC developmental signal from BCL6.

A study by He et al. revealed that the class switching process preferentially occurs in late G1 to early S phase, and is mediated by the high kinase activity of CDK2 during that period (He et al., 2015). CDK2 recruits the activation-induced cytidine deaminase (AID), a key regulator of class switch recombination, to a switch region DNA (He et al., 2015). However, it remains unclear how CDK2 promotes AID nuclear accumulation, whether the regulation is cell-cycle-independent, and whether AID is a CDK2 substrate.

### Direct Roles of CDK2 and CDK Inhibitor p27^KIP1^ in T cell Anergy

Several lines of evidence indicate that CDK2 and CDK2 inhibitor p27^KIP1^ represent key switches for T cell anergy. Naïve T cells require signals from co-stimulatory receptor CD28. Upon co-stimulation, T cells undergo massive cell expansion, and become functionally activated. However, if T cells do not receive appropriate co-stimulation in the presence of specific antigen recognition or when CD28 signaling is blocked, T cell activity is diminished, a process known as anergy. T cell anergy is a primary mechanism for peripheral tolerance, which is critical for preventing reactivity against self-antigens or allograft rejection. Recently, the roles of CDK2 and p27^KIP1^ in T cell anergy have been extensively reviewed (Wells and Morawski, 2014).

**FIGURE 2:**
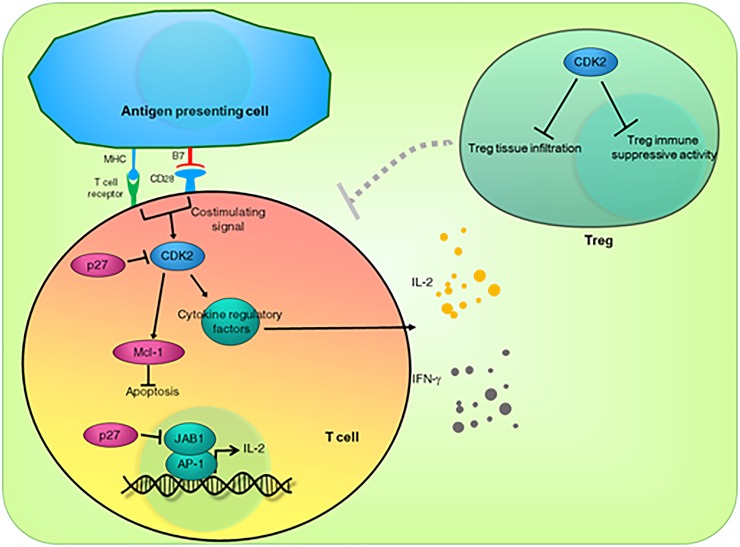
Direct roles of CDK2 and CDK inhibitor p27^KIP1^ in T cell anergy. CDK2 is a mediator for co-stimulating signals. CDK2 activity is required for the activity of antigen-activated T cells. CDK2 may contribute to activated T cell survival by upregulating the anti-apoptotic protein Mcl-1. p27^KIP1^ inhibits CDK2 activity, which in turn acts as an “off” switch for activated T cells. In addition, independent of its CDK2-inhibitory function, p27^KIP1^ sequesters JAB-1 from the AP-1 transcriptional complex, leading to downregulated IL-2 expression. CDK2 also inhibits Treg activity and tumor infiltration, further de-repressing the function of immune cells.

In mouse models, depletion of CDK2 or inhibition of CDK2 activity by small molecule inhibitors in allograft recipients reduce allograft rejection by fivefold and promote long-term allograft retention. It was shown that CDK2-deficient T cells proliferate normally, but have a marked decrease of IL-2 and type II IFN (IFN-γ) (Chunder et al., 2012). This finding indicates that CDK2 helps mediate the co-stimulatory CD28 signaling (Figure 2). It is unclear how CDK2 activity promotes the activity of the activated peripheral T cells. However, it is likely that CDK2 directly phosphorylates and regulates several regulatory proteins controlling cytokine gene expression. SMAD3, JUN, SP1, STATs, and EZH2 are among the known direct substrates for CDK2 (Nellore et al., 2014; Wells and Morawski, 2014). An alternative explanation is that CDK2 activity is required for expression of the anti-apoptotic protein Mcl-1 (Figure 2). Inhibition of CDK2 activity rapidly downregulates T cell Mcl-1 protein, which has a short half-life, leading to T cell apoptosis (Li et al., 2009).

In contrast to the positive roles of CDK2 in T cell activity, p27^KIP1^ expression blocks CD28 signaling and promotes T cell anergy even in co-stimulated T cells. The CDK2-inhibitory function of p27^KIP1^ may contribute to this outcome. In addition, p27^KIP1^ associates with the c-Jun co-activator JAB1, resulting in the CDK2-independent reduced transactivation of AP-1 and IL-2 transcription (Boussiotis et al., 2000; Rowell et al., 2006), providing an extra “off” switch for co-stimulated T cell activity (Figure 2).

Regulatory T cells (Tregs) suppress other cells in the immune system. Interestingly, CDK2 deficiency promotes Treg tissue infiltration and suppresses immune activity (Chunder et al., 2012). According to these findings, inhibition of CDK2 or overexpression of p27^KIP1^ should promote self-tolerance and alloantigen tolerance. On the other hand, hyperactive CDK2 or suppressed p27^KIP1^ expression should promote autoimmune disease or allograft rejection. Therefore, pharmacological inhibition of CDK2 may be beneficial for transplant patients, alone or combined with co-stimulatory CD28-inhibiting agents (Figure 2).

A drug inhibiting CDK2 was recently shown to block alloimmune pathology in a murine model of graft-vs-host disease (GVHD) (Li et al., 2009). In addition, recent studies demonstrated that Roscovitine (a potent small molecule inhibitor of CDK2, and weak inhibitor of CDK1, 5, 7, and 9) treatment inhibits the function of the alloreactive T cells while allowing preservation of leukemia-specific and pathogen-specific effectors (Nellore et al., 2014). These findings support CDK2 inhibition as a novel approach for GVHD management. Further work is required to identify the precise mechanism(s) by which CDK2 inhibition mediates the preservation and the increase of pathogen-specific and leukemia-specific effectors, while suppressing activation and expansion of alloreactive effectors, as well as to confirm these results *in vivo*.

### Role of CDK4/6 in Tumor Immune Tolerance Revealed by CDK4/6 Inhibitor Treatment

In the past, most of the studies about immunomodulatory effects of cell cycle regulators have examined genetically engineered knockout mice. Gene ablation may force cells to compensate for the loss, thereby obscuring the true function of a given protein. Thus, the systemic functions of cell cycle regulators in the immune system should be studied *in vivo*, where CDK activity can be shut down after the immune system has fully developed by using specific small molecule inhibitors.

A series of recent independent investigations has demonstrated a critical cell cycle-independent function of CDK4/6 in tumor immunosurveillance using small molecule CDK4/6 inhibitors (Palbociclib, Ribociclib, or Abemaciclib).

PD-L1 is a surface antigen expressed on normal and tumor cell membranes. This ligand is believed to mediate suppression of immune cells by inducing T cell exhaustion. Binding of PD-L1 to PD-1 on T cells transmits an inhibitory signal that reduces the proliferation of antigen-specific T cells. When cancer cells express high levels of PD-L1, they induce immune tolerance to the tumor. Blockage of PD-L1 stimulates effector T cells to produce antitumor responses. Therefore, cancer cells with high PD-L1 expression provide a good target for PD-L1 inhibition by immune checkpoint inhibitors (ICIs) (Alsaab et al., 2017).

**FIGURE 3:**
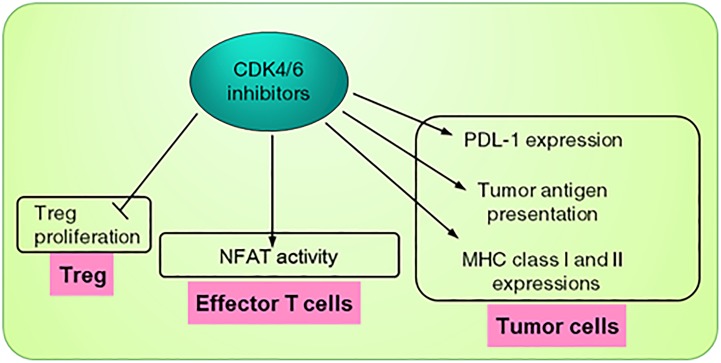
Roles of CDK4/6 inhibitor in tumor immunosurveillance. CDK4/6 inhibitor exerts tumor immunomodulation via 3 types of cells. In tumor cells, the inhibitor promotes expression of PDL-1, causing the tumor to be vulnerable to the immune checkpoint inhibitor (ICI). CDK4/6 inhibitor also promotes expression of MHC I/II and increases tumor antigen presentation. In effector T cells, CDK4/6 inhibitor increases activity of NFATs, key regulators of T-cell development and function. Lastly, CDK4/6 inhibitor indirectly promotes immune function by inhibiting Treg proliferation.

PD-L1 abundance was found to be directly regulated *via* cyclin D-CDK4 kinase activity (Zhang et al., 2018). Inhibition of CDK4 by small molecule inhibitors or by p16^INK4A^ expression upregulates and stabilizes PD-L1 even in RB1-deficient cells, indicating that this is cell cycle-independent. PD-L1 is kept low by SPOP-mediated proteasome degradation. Cyclin D-CDK4 phosphorylates SPOP at Ser6 and increases SPOP abundance, leading to reduction in the level of PD-L1. Therefore, treatment by the small molecule CDK4/6 inhibitor lowers SPOP and promotes PD-L1 protein expression, priming tumor cells for ICI treatment. In an independent study, Goel et al. (2017) showed that blocking CDK4/6 activity promotes cancer cell expression of endogenous retroviral elements, leading to enhanced tumor antigen presentation, demonstrating a different mechanism for T cell response. In agreement with that, Schaer et al. (2018) reported an upregulation of select chemokines (MIP1α, MIP1β), iNOS, PD-L1, and PD-L2 in tumors treated with high dose of Abemaciclib. In addition, combination of abemaciclib with anti-PD-L1 resulted in a markedly tumor inflamed phenotype shown by greater than twofold upregulation of multiple T cell activation-associated genes encoding IFNγ, co-inhibitory receptors and ligands (PD-1, PD-L1, PD-L2, LAG3, VISTA), cytokines and chemokines (IL10, TNFα, MIP1α, MIP1β, RANTES, GRO), and IFNγ-dependent enzymes (IDO1, iNOS). In addition, they showed that CDK4/6 inhibitor treatment causes MHC class I and II upregulation in tumor cells (Schaer et al., 2018; Figure 3).

The CDK4/6 inhibitor not only acts on tumor cells but also acts indirectly and directly on the immune cells responsible for antitumor immunity. First, the CDK4/6 inhibitor suppresses proliferation of Treg and relieves Treg inhibition on effector T cells (Goel et al., 2017; Figure 3). Second, inhibition of CDK4/6 decreases the repression of NFAT family proteins and their target genes, critical regulators of T cell function, which in turn increases tumor infiltration and activity of effector T cells (Figure 3). Therefore, blocking CDK4/6 activity on T cells facilitates the cytotoxic activity of T cells to kill tumor cells (Deng et al., 2018; Schaer et al., 2018). Since the activity of the adaptive immune cells can be regulated by the innate counterpart, it may be important to carefully examine the consequence of CDK4/6 inhibition on innate immune cells such as macrophages and dendritic cells in tumor microenvironment. In fact, gene expression analysis of a tumor treated with a combination of Abemaciclib and anti-PD-L1 showed enrichment of genes in dendritic cell (DC) maturation; this observation indicates that combination treatment may directly or indirectly affect immunological events beyond T cell activation and may modulate innate immune mechanisms resulting in enhanced antigen presentation and T cell priming (Schaer et al., 2018).

It has been verified that CDK4/6 inhibition enhances the response to PD-1 blockade in an *ex vivo* organotypic tumor spheroid culture system; *in vivo*, CDK4/6 inhibition enhances tumor regression and markedly improves overall survival rates of murine syngeneic models (Deng et al., 2018; Zhang et al., 2018).

Recently, Jerby-Arnon et al. (2018) performed single cell RNA sequencing of more than 7,000 cells from 33 melanoma tumors, comprised of both ICI-responsive and ICI-resistant tumors. They identified the “T cell Exclusion Program,” a set of mRNAs induced or repressed in ICI-resistant melanoma. A similar Exclusion Program was also detected in acquired ICI-resistant melanoma. Interestingly, CDK4 and its downstream targets were among the induced mRNAs, and inhibition of CDK4 activity by a small molecule CDK4/6 inhibitor reinstated the malignant cell population to a less immune-resistant state. Together, these results indicate that CDK4/6 are master modulators of ICI responsiveness.

While the connection between CDK4/6 activity and antitumor T cell function has been established by several seminal reports, it remains unclear whether this role of CDK4/6 exists in non-cancer contexts. It is uncertain why cells with limited CDK4/6 activity should be protected from T cells, and why cells with high CDK4/6 activity should be under close T cell surveillance. It may be that cells with high CDK4/6 activity are usually maintained in the highly proliferative stage and are prone to genetic damage, therefore, they may need to be quickly removed from the tissue by T cells. Further investigations in non-cancer contexts are needed.

Collectively, these results strongly support the notion that, in cancer, CDK4/6 inhibition not only halts the cancer cell cycle but also promotes T cell-dependent anti-tumor activities in combination with ICI. Because of the strong clinical potential of these findings, CDK4/6 inhibitors are being combined with checkpoint inhibitors (PD-1 or PD-L1 inhibitors) in several clinical studies in non-small cell lung cancer, breast cancer, and squamous cell carcinoma (PACE [NCT03147287], NCT02778685, NCT02778685, NCT03655444, and NCT02779751^1^).

## Summary and Outlook

Recent research provides cumulative evidence for additional functions of cell cycle regulators in several biological processes, including DNA damage repair, cell death, cell differentiation, metabolism, and immune defense. Some of these functions may be related to cell division. However, it is unclear why cell cycle regulators have roles outside cell cycle regulation, especially systemic roles like immunomodulation. The immunomodulatory functions of cell cycle regulators are unique among the non-cell cycle functions, as this focuses on cell-cell, cell-environment, or cell-pathogen interactions in multicellular organisms.

Overall, cell cycle regulators orchestrate the cell cycle with optimal immune functioning. Often, they directly phosphorylate protein regulators of immune function. Interfering with CDK activity by small molecule inhibitors or genetic deletion results in noticeable immunological outcomes, such as weakened immunity, autoimmunity, or immunotolerance, signifying that this novel role of cell cycle regulators is essential for whole body homeostasis, and is clinically relevant.

Many of these studies were carried out in *in vitro* models and are able to detect a limited set of surrogate outcomes. Therefore, the initial understanding of this novel role of cell cycle regulators is still fragmented, and scarcely represents the systemic contribution of cell cycle regulators to the immune system. Further investigation surrounding the *in vivo* models is required. In particular, long-term observation on immune functions in immunocompetent animals treated with small molecule CDK inhibitors is warranted.

The non-canonical role of cell cycle regulators in immune cell modulation is of interest due to its clinical relevance. Further study of this fascinating role of cell cycle regulators is not only essential for understanding basic cell cycle biology but may open new avenues for treating diseases such as autoimmune diseases and cancer.

## Author Contributions

SJ planned and wrote the manuscript. PL wrote the manuscript. Both authors approved the final manuscript.

## Conflict of Interest Statement

The authors declare that the research was conducted in the absence of any commercial or financial relationships that could be construed as a potential conflict of interest.

## Footnotes

1 https://clinicaltrials.gov
